# High-specificity identification of large vessel occlusion stroke using D-dimer and NT-proBNP combined with clinical variables

**DOI:** 10.3389/fneur.2026.1830051

**Published:** 2026-06-24

**Authors:** Leire Azurmendi, J. Gonzalez-Fraile, A. Barragán-Prieto, S. Reymond, R. M. Delgado, C. de Jesús-Gil, A. Penalba, J. A. Cabezas-Rodríguez, F. Moniche, S. Pérez-Sánchez, A. González-Garcia, J. Montaner, J. C. Sanchez

**Affiliations:** 1 Department of Internal Medicine, Faculty of Medicine, Geneva University, Genève, Switzerland; 2 ABCDx SL, Barcelona, Spain; 3 Neurovascular Research Group, Institute of Biomedicine of Seville, IBiS/Virgen Macarena University Hospital/CSIC/University of Seville, Seville, Spain; 4 Neurovascular Research Laboratory, Vall d'Hebron Institute of Research, Hospital Vall d'Hebron, Universitat Autònoma de Barcelona, Barcelona, Spain; 5 Neurology Clinical Management Unit, Institute of Biomedicine of Seville, IBiS/Virgen del Rocío University Hospital/CSIC/University of Seville, Seville, Spain

**Keywords:** acute ischemic stroke, blood biomarkers, clinical scales, D-dimer, large vessel occlusion, multivariable panels, NT-ProBNP, prehospital triage

## Abstract

**Background:**

Rapid identification of large vessel occlusion (LVO) is essential to optimize prehospital triage and timely access to mechanical thrombectomy, yet current clinical scales show limited diagnostic performance, particularly when high specificity is required to ensure patient safety.

**Methods:**

We performed a prospective analysis of 290 consecutive patients with suspected acute ischemic stroke presenting within 24 h of symptom onset (*n* = 96 LVO), including 223 patients presenting within 6 h (*n* = 71 LVO). Presence of LVO was determined by vascular neuroimaging (computed tomography angiography or magnetic resonance angiography) performed during routine clinical care. Circulating blood biomarkers and clinical variables were systematically evaluated to derive a multivariable panel for LVO detection. A data-driven modeling strategy (PanelomiX) was applied under predefined high-specificity constraints, with repeated stratified cross-validation used for panel ranking and robustness assessment. Diagnostic performance was compared against individual variables and previously reported biomarker–clinical combinations.

**Results:**

A multimodal panel combining NT-proBNP, D-dimer, mean blood pressure, and the National Institutes of Health Stroke Scale (NIHSS) score demonstrated superior discrimination compared with individual biomarkers, clinical variables alone or any other considered biomarker-clinical combination. At a specificity of 90%, the panel achieved a sensitivity of 59.8% in the 24 h cohort. Among patients presenting within 6 h of symptom onset, sensitivity reached 65.7% while maintaining a specificity of 90.3%. Cross-validated Youden index estimates decreased to 0.44 in both cohorts, confirming modest optimism bias consistent with the exploratory design of this single-cohort study.

**Conclusion:**

The integration of circulating biomarkers with clinical variables improved the high-specificity identification of LVO. This multimodal approach should be interpreted as a rule-in enrichment strategy to support early triage and optimized routing of patients with a high probability of LVO, rather than as a rule-out tool to exclude LVO in lower-risk patients.

## 1 Introduction

Large vessel occlusion (LVO) is a major determinant of severe disability and mortality in acute ischemic stroke. Although LVO represents only a minor portion of ischemic strokes, it contributes disproportionately to stroke-related morbidity and long-term functional impairment (1). Mechanical thrombectomy has dramatically improved outcomes in this population; however, its efficacy is strongly time-dependent, with treatment delays reducing the probability of functional independence (2, 3). Early and accurate identification of LVO is therefore essential.

Definitive diagnosis of LVO relies on imaging, including computed tomography angiography or magnetic resonance angiography, modalities that are only available in hospital settings and MSUs (4, 5). Consequently, patients initially transported to centers without thrombectomy capability frequently require secondary interhospital transfer, leading to treatment delays and poorer outcomes. Optimizing pre-hospital triage to enable direct transport of appropriate patients to comprehensive stroke centers remains a critical challenge in current stroke systems of care.

To address this gap, several prehospital clinical severity scales have been developed to estimate the likelihood of LVO and guide routine decisions. Despite widespread implementation, their diagnostic performance is inconsistent, and no single scale reliably achieves both high sensitivity and high specificity across diverse clinical environments (6, 7). This limitation is particularly problematic in triage strategies that require high specificity to avoid unnecessary bypass of primary stroke centers and inefficient resource utilization.

These constraints highlight the need for complementary objective tools to support early decision-making. Circulating blood biomarkers reflecting key pathophysiological mechanisms implicated in LVO, such as coagulation activation (D-dimer), cardiac dysfunction (NT-proBNP), and neuronal or glial injury (e.g., GFAP, H-FABP), have emerged as promising candidates (8–17). However, when assessed individually, most biomarkers have shown insufficient accuracy for standalone use in prehospital triage (2, 18–20).

Given the multifactorial and heterogeneous nature of LVO pathophysiology, it is unlikely that a single biomarker or clinical variable can provide adequate discrimination. Integrating complementary biological and clinical information may better capture the underlying disease complexity and improve diagnostic performance in time-critical constraints. In particular, the evaluation of multimodal panels under predefined high-specificity conditions reflects real-world triage requirements.

In this study, we evaluated the diagnostic performance of selected circulating biomarkers, individually, and in combination with clinical variables, for the identification of LVO in patients with suspected acute ischemic stroke. We hypothesized that a multivariable biomarker–clinical panel would improve LVO detection while maintaining high specificity, thereby supporting early triage decisions within established stroke care pathways.

## 2 Materials and methods

### 2.1 Study design and cohort description

This study represents a secondary analysis of prospectively collected clinical data and biological samples obtained within the BIOFAST study, a multicenter observational study designed to investigate circulating biomarkers in patients with suspected acute stroke in the prehospital setting.

BIOFAST prospectively enrolled consecutive adult patients with suspected acute stroke attended by the emergency medical services (EMS) and transported to participating stroke centers in Seville, Spain.

The study was conducted in collaboration with the Andalusian Emergency Medical Service (EPES) and the stroke units of participating tertiary hospitals. Details of the study design and procedures are described in the clinical protocol.

### 2.2 Study population

Patients were eligible if they presented with symptoms compatible with acute stroke within ≤24 h from symptom onset or last known well, according to the predefined study protocol.

For the present analysis, we included patients with available clinical data, biomarker measurements, and definitive vascular imaging. To increase statistical power for biomarker panel discovery, the dataset was expanded beyond the strictly prospective BIOFAST inclusion window to include additional patients recruited within the same clinical research network and time period using comparable procedures.

In total, 290 consecutive patients with suspected acute ischemic stroke presenting within 24 h from symptom onset were included in the main analysis cohort. Among them, 223 patients presented within 6 h, corresponding to the prospective BIOFAST inclusion criteria.

### 2.3 Outcome definition

The primary endpoint was the presence of large vessel occlusion (LVO) identified by vascular neuroimaging (computed tomography angiography or magnetic resonance angiography) performed during routine clinical care.

LVO was defined as occlusion of a major intracranial artery, including the internal carotid artery, basilar artery, or proximal segments of the middle cerebral artery (M1 segment or proximal M2). Patients were dichotomized into LVO and non-LVO groups. The non-LVO group intentionally included ischemic strokes without LVO, intracerebral hemorrhage, and stroke mimics, because prehospital triage decisions are made before definitive imaging-based diagnosis. Restricting the comparator group to ischemic non-LVO strokes only would therefore represent an artificial scenario with limited operational relevance.

### 2.4 Clinical variables

Clinical variables were collected at first medical assessment.

Stroke severity was evaluated using the National Institutes of Health Stroke Scale (NIHSS) at hospital admission, and the Cincinnati Prehospital Stroke Scale was recorded during prehospital evaluation when available.

Vital signs including systolic and diastolic blood pressure, heart rate, and blood glucose were recorded according to routine emergency care procedures, as well as age and sex of the patient.

Mean arterial pressure was calculated using the standard formula (SBP + 2 × DBP) / 3 from the first recorded blood pressure measurement.

### 2.5 Marker measurement

Peripheral whole blood was obtained as early as possible after first medical contact. Samples were centrifuged at 1500 × g for 15 min at 4 °C to obtain EDTA plasma, which was subsequently stored at −80 °C until analysis.

Plasma concentrations of H-FABP, NT-proBNP, and GFAP were measured using R-PLEX electrochemiluminescence immunoassays on the Meso Scale Discovery (MSD) platform (MSD, Gaithersburg, MD, United States). Samples were randomly distributed across plates, and assays were performed according to the manufacturer’s instructions. A control sample was included in triplicate on each plate to calculate the inter-plate coefficient of variation (CV).

D-dimer levels were measured using assays available for routine clinical use, specifically the STA-Liatest D-Di immunoturbidimetric assay on STA-R Evolution or STA Compact analyzers (Diagnostica Stago, Asnières-sur-Seine, France), according to the manufacturer’s instructions.

### 2.6 Ethics statement

The study was conducted in accordance with the principles of the Declaration of Helsinki. The study protocol was approved by the Comité de Ética de la Investigación of Hospital Universitario Virgen Macarena y Virgen del Rocío (Seville, Spain) (protocol code DTS19/00135). The study was registered at ClinicalTrials.gov (identifier NCT04612218). Written informed consent was obtained from all participants or their legal representatives according to local regulatory requirements. Biological sample collections linked to the project were registered with the Instituto de Salud Carlos III, including collection C.0005032 (Instituto de Biomedicina de Sevilla, IBIS) and collection C.0003176 (Hospital Vall d’Hebron).

### 2.7 Statistical data analysis

All statistical analyses were performed using R version 4.4.2 (R Foundation for Statistical Computing, Vienna, Austria) within RStudio (version 2024.12.1). Patients were dichotomized into two groups: (1) no LVO and (2) LVO. Continuous variables were assessed for normality using the Shapiro–Wilk test. As biomarker levels were non-normally distributed (*p* < 0.05), group comparisons were performed using the nonparametric Mann–Whitney U test. Categorical variables were compared using the chi-squared test or Fisher’s exact test, as appropriate. A two-sided *p*-value < 0.05 was considered statistically significant. The discriminative performance of individual biomarkers was evaluated using receiver operating characteristic (ROC) curve analysis with the pROC package in R. The area under the ROC curve (AUC) and corresponding 95% confidence intervals were calculated. For each biomarker, operating points were selected by maximizing sensitivity under a predefined constraint of ≥ 90% specificity. Given the exploratory nature of this analysis, biomarker panel identification and performance evaluation were conducted within the same cohort without a separate derivation and validation dataset.

### 2.8 Panel development

Multivariable panel selection was performed using the PanelomiX software (21), which applies the Iterative Combination of Biomarkers and Thresholds (ICBT) algorithm. Panel optimization prioritized high specificity (≥90%) while maximizing sensitivity within this constraint, consistent with the intended clinical role of the model as a prehospital triage enrichment tool for selected direct-routing decisions rather than a universal LVO screening or exclusion strategy. This design inherently favors specificity over sensitivity and therefore does not aim to identify all LVO patients.

Candidate variables included circulating biomarkers (NT-proBNP, D-dimer, GFAP, H-FABP) and clinical parameters (age, sex, NIHSS, mean blood pressure, and glucose). Panel size was restricted to a maximum of four variables to limit model complexity, reduce overfitting risk, and preserve potential clinical applicability in time-critical settings. The ICBT algorithm systematically evaluates all possible combinations of candidate variables and corresponding decision thresholds. We implemented a repeated stratified cross-validation scheme (10-fold, 5 repeats) for panel ranking. The average performance across validation folds was used to rank the panels and mitigate optimism bias, ranking was based on Youden performance metric. Thresholds were always derived from the full dataset. The cross-validation scheme was also used to assess the relative robustness of performance results.

Biomarker data were not available for all patients. Missing biomarker values were 0.7% for D-dimer, 1% for NT-proBNP, 3.1% for H-FABP, and 15.9% for GFAP. To maximize data retention while maintaining panel-specific consistency, missing values were handled using an available-case approach: patients were excluded only when lacking data for a variable included in the specific panel under evaluation. No imputation was performed. Although all possible combinations were explored algorithmically, only the best-performing panels and those with clinical plausibility are presented. Atrial fibrillation was excluded as a candidate panel variable due to pre-hospital limitations: it was recorded as a pre-existing medical history variable in this study, making it subject to availability and recall limitations inherent to acute prehospital assessment. Conceptually, it represents a competing etiological mechanism rather than an independent diagnostic marker for LVO. Diabetes history was excluded as redundant with point-of-care glucose, which provides a more direct and precise representation of acute metabolic status than a binary history label.

## 3 Results

### 3.1 Demographic and clinical characteristics

Table 1 summarizes the baseline demographic and clinical characteristics. The study cohort included 290 patients with suspected acute ischemic stroke, of whom 96 (33.1%) were diagnosed with LVO. Among the 290 included patients, 191 were diagnosed with ischemic stroke, including 96 cases with LVO and 95 non-LVO ischemic strokes, while 51 patients presented intracerebral hemorrhage and 48 were classified as stroke mimics. Sex distribution differed significantly between groups (*p* = 0.02), and patients with LVO were significantly older than those without LVO (76 [47, 95] vs. 70 [34, 93],*p* = 0.003). Stroke severity at admission, assessed by the NIHSS score, was also higher in the LVO group (15 [1, 35] vs. 5 [0, 35], *p* < 0.001).

**Table 1:** Demographic and clinical characteristics of the study population according to LVO status (≤24 h from symptom onset).

Clinical and demographic characteristics of patients with and without large vessel occlusion				
	No LVO	LVO	Overall	*p*-value
	(*N* = 194)	(*N* = 96)	(*N* = 290)	
Age (years)				
Mean (SD)	69.7 (12.0)	74.9 (10.5)	71.4 (11.8)	0.003
Median [Min, Max]	70.0 [34.0, 93.0]	76.0 [47.0, 95.0]	73.0 [34.0, 95.0]	
Sex				
Male	124 (63.9%)	45 (46.9%)	169 (58.3%)	0.022
Female	70 (36.1%)	51 (53.1%)	121 (41.7%)	
NIHSS				
Mean (SD)	8.46 (8.99)	14.8 (7.25)	10.6 (8.94)	<0.001
Median [Min, Max]	5.00 [0, 35.0]	15.0 [1.00, 35.0]	8.00 [0, 35.0]	
Systolic blood pressure (mmHg)				
Mean (SD)	154 (32.4)	153 (24.4)	154 (29.9)	0.976
Median [Min, Max]	152 [80.0, 260]	150 [96.0, 221]	151 [80.0, 260]	
Diastolic blood pressure (mmHg)				
Mean (SD)	85.5 (19.1)	83.5 (12.5)	84.8 (17.2)	0.697
Median [Min, Max]	86.0 [34.0, 171]	83.0 [55.0, 114]	85.0 [34.0, 171]	
Mean blood pressure (mmHg)				
Mean (SD)	100 (34.9)	98.8 (30.7)	99.9 (33.5)	0.893
Median [Min, Max]	106 [0, 182]	106 [0, 150]	106 [0, 182]	
Diabetes mellitus				
No	125 (64.4%)	71 (74.0%)	196 (67.6%)	0.265
Yes	69 (35.6%)	25 (26.0%)	94 (32.4%)	
Atrial fibrillation				
No	160 (82.5%)	70 (72.9%)	230 (79.3%)	0.167
Yes	34 (17.5%)	26 (27.1%)	60 (20.7%)	
Heart rate (beats/min)				
Mean (SD)	81.0 (17.6)	79.3 (16.5)	80.4 (17.2)	0.864
Median [Min, Max]	78.0 [35.0, 140]	77.0 [44.0, 140]	78.0 [35.0, 140]	
Glucose (mg/dL)				
Mean (SD)	138 (66.8)	136 (59.1)	138 (64.3)	0.862
Median [Min, Max]	121 [66.0, 750]	117 [76.0, 382]	119 [66.0, 750]	
Smoking				
Former smoker	56 (28.9%)	19 (19.8%)	75 (25.9%)	0.597
Never smoker	103 (53.1%)	58 (60.4%)	161 (55.5%)	
Current smoker	35 (18.0%)	19 (19.8%)	54 (18.6%)	

Data are presented as mean (SD), median [min–max], or number (%), as appropriate. *p*-values were calculated using Student’s *t*-test, Mann–Whitney U test, or *χ*^2^ test, as appropriate.

No significant differences between groups were observed for systolic or diastolic blood pressure, heart rate, glucose levels, diabetes mellitus, atrial fibrillation, smoking status (all *p* > 0.05).

### 3.2 Circulating biomarker levels in LVO and no LVO patients

Circulating concentrations of NT-proBNP, D-dimer, H-FABP and GFAP were compared between patients with and without large vessel occlusion. Detailed values are summarized in Table 2.

**Table 2:** Circulating biomarker concentrations in patients with and without large vessel occlusion.

Variable	No LVO	LVO	Overall	*p*-value
	(*N* = 194)	(*N* = 96)	(*N* = 290)	
H-FABP (ng/mL)				
Mean (SD)	11.6 (25.4)	11.7 (19.4)	11.6 (23.6)	0.479
Median [Min, Max]	6.96 [1.21, 244]	7.53 [1.52, 161]	7.12 [1.21, 244]	
NT-proBNP (pg/mL)				
Mean (SD)	3,120 (20800)	2,220 (3540)	2,820 (17200)	0.014
Median [Min, Max]	535 [11.7, 285,000]	1,140 [55.9, 20,600]	688 [11.7, 285,000]	
D-dimer (ng/mL)				
Mean (SD)	932 (689)	1,210 (661)	1,020 (691)	<0.001
Median [Min, Max]	642 [106, 2,280]	1,160 [187, 2,280]	785 [106, 2,280]	
GFAP (pg/mL)				
Mean (SD)	2090 (11800)	118 (221)	1,410 (9600)	0.976
Median [Min, Max]	61.1 [0.333, 131,000]	70.9 [0.217, 1840]	67.4 [0.217, 131,000]	

NT-proBNP concentrations were significantly higher in patients with LVO compared with those without LVO (median 1,140 pg./mL vs. 535 pg./mL, *p* = 0.014). Similarly, D-dimer levels were significantly elevated in the LVO group (median 1,160 ng/mL vs. 642 ng/mL, *p* < 0.001). In contrast, no statistically significant differences were observed between groups for H-FABP or GFAP concentrations.

### 3.3 Prediction of large vessel occlusion using individual biomarkers and clinical parameters

To assess the discriminative performance of individual clinical variables and circulating biomarkers for LVO detection, sensitivity and specificity were calculated (Table 3). Both variables were calculated under a predefined high-specificity constraint (≥90%), reflecting the requirements of prehospital triage where false-positive referrals must be minimized (Table 3). Under this constraint, overall sensitivity remained low across all individual predictors. Among clinical variables, age and NIHSS achieved the highest sensitivities. Age reached a sensitivity of 20% at 91% specificity (cut-off 85.5 years), while NIHSS reached 17% sensitivity at 90% specificity (cut-off 21.5).

**Table 3:** Diagnostic performance of individual clinical variables and circulating biomarkers for large vessel occlusion under high-specificity constraints.

Predictor	SE, % (95% CI)	SP, % (95% CI)	Cut-off
Age	19.8 (13.1–28.9)	91.2 (86.4–94.5)	85.5
NIHSS score	16.7 (10.5–25.4)	90.3 (85.1–93.8)	21.5
NT-proBNP (pg/mL)	13.7 (8.2–22.0)	90.1 (85.1–93.6)	3725.55
H-FABP (ng/mL)	12.0 (6.8–20.2)	90.5 (85.4–93.9)	17.85
D-dimer (ng/mL)	10.5 (5.8–18.3)	94.3 (90.1–96.8)	2231.39
Heart rate (beats/min)	9.0 (4.4–17.4)	93.9 (88.8–96.8)	59
Glucose (mg/dL)	6.5 (3.0–13.5)	91.0 (86.1–94.3)	90.5
Systolic blood pressure (mmHg)	4.5 (1.8–11.0)	90.0 (84.7–93.6)	114.5
Mean blood pressure (mmHg)	2.1 (0.6–7.3)	90.2 (85.2–93.6)	132.5
GFAP (pg/mL)	1.2 (0.2–6.4)	90.0 (84.4–93.8)	1439.9
Diastolic blood pressure (mmHg)	1.1 (0.2–6.1)	93.3 (88.7–96.1)	59
Cincinnati Prehospital Stroke Scale	0.0 (0.0–4.0)	100.0 (98.0–100.0)	Inf

SE = sensitivity; SP = specificity.

Among circulating biomarkers, NT-proBNP and H-FABP showed sensitivities of 14 and 12%, respectively, at 90% specificity. D-dimer and heart rate reached higher specificity values (94%), but with lower sensitivities (11 and 9%, respectively). Glucose demonstrated a sensitivity of 7% at 91% specificity. Blood pressure parameters (systolic, mean, and diastolic) and GFAP exhibited sensitivities below 5% despite maintaining ≥90% specificity. For the Cincinnati scale, no possible threshold led to specificities above 90%, beyond the trivial case with 100% specificity and 0% sensitivity – that failed to identify any LVO cases under such threshold. Only reducing the constraint on the specificity led to non-trivial cases --e.g. with a specificity of 70.6% for a sensitivity of 58.9% (cut-off 2.5).

Overall, no single biomarker or clinical variable provided sufficient sensitivity at high specificity, supporting the rationale for multivariable panel development.

### 3.4 Panel selection

#### 3.4.1 Identification of the optimal multivariable panel

Given the limited discriminative performance of individual variables, we evaluated whether combining circulating biomarkers with clinical parameters improved LVO classification. Candidate biomarkers (NT-proBNP, D-dimer, H-FABP and GFAP) and clinical variables (age, sex, mean blood pressure, glucose, NIHSS, Cincinnati scale) were systematically combined into all possible panels of up to 4 variables with the only condition that NIHSS or Cincinnati scale must be in the panel. Panels were developed using the PanelomiX ICBT algorithm, with the ranking based on the Youden performance computed within a stratified repeated cross-validation scheme.

The best-performing panel consisted of NT-proBNP, D-dimer, mean blood pressure, and NIHSS. The derived decision rule applied the following thresholds: NT-proBNP > 472.0 pg./mL, D-dimer > 399.4 ng/mL, mean blood pressure > 79.5 mmHg, and NIHSS ≥ 8. A case was classified as LVO when all four criteria were met.

This model achieved a sensitivity of 59.8% at a specificity of 90% for the 24 h cohort (Table 4). Supplementary Figure S2 illustrates the relative diagnostic performance of individual predictors and the final multimodal panel under fixed high-specificity conditions. This multimodal panel was ranked the highest within the implemented repeated stratified cross-validation scheme (Supplementary Table S1). Its cross-validated Youden index estimate was 0.44, suggesting only modest optimism bias, consistent with the exploratory design of this single-cohort study. The cross-validated ROC AUC for the model was 0.76, with a normalized cross-validated partial ROC-AUC of 0.70, defined for the region with specificities above 80%.

**Table 4:** Diagnostic performance and thresholds of the optimal clinical-only panel in the 24-h cohort, its application in the 6-h population, and substitution of NIHSS with the Cincinnati prehospital stroke scale.

Panel components: NT-proBNP, D-dimer, mean blood pressure, NIHSS/Cincinnati	6-h			24-h		
	SE, % (95% CI)	SP, % (95% CI)	Thresholds	SE, % (95% CI)	SP, % (95% CI)	Thresholds
NIHSS	65.7 (55.2–76.2)	90.3 (85.5–95.1)	NT-proBNP > 564.1 pg./mL; D-dimer > 399.4 ng/mL; mean blood pressure > 79.5 mmHg; NIHSS ≥ 8; panel positive when ≥4 criteria are met Youden: 0.56	59.8 (49.3–70.3)	90 (85.2–94.8)	NT-proBNP > 472.0 pg./mL; D-dimer > 399.4 ng/mL; mean blood pressure > 79.5 mmHg; NIHSS ≥ 8; panel positive when ≥4 criteria are met Youden: 0.50
Cincinnati	56.1 (45.6–66.6)	90.4 (85.6–95.2)	NT-proBNP > 165.35 pg./mL; D-dimer > 512.5 ng/mL; mean blood pressure < 129 mmHg; Cincinnati > 2.5; panel positive when ≥4 criteria are met Youden: 0.44	51.2 (40.7–61.7)	90.1 (94.9–95.3)	NT-proBNP > 143.4 pg./mL; D-dimer > 923.1 ng/mL; mean blood pressure <121 mmHg; Cincinnati > 1.5; panel positive when ≥4 criteria are met Youden: 0.37

Sensitivity and specificity are reported at the predefined high-specificity threshold. SE = sensitivity; SP = specificity.

The second best-ranked panel incorporated H-FABP but demonstrated inferior cross-validated performance and was therefore not retained. Cross-validated performance metrics for all evaluated panels are provided in Supplementary Table S1.

#### 3.4.2 Comparison with clinical-only panel configurations

To determine the added value of biomarker integration, multivariable panels restricted to clinical parameters were evaluated in the 24-h cohort. The clinical-only model combining NIHSS and mean blood pressure, thus excluding the circulating biomarkers from the best panel above, achieved a sensitivity of 32.9% at a specificity of 91.1% for the 24-h cohort. The best-performing clinical-only model combined only two variables, NIHSS and glucose, achieving a sensitivity of 43.5% at a specificity of 90.6% for the 24-h cohort (Table 5).

**Table 5:** Diagnostic performance of the best clinical-only panel and the clinical component of the optimal multimodal panel in the 6-h cohort and 24-h cohort.

Panel components	6-h			24-h		
	SE, % (95% CI)	SP, % (95% CI)	Thresholds	SE, % (95% CI)	SP, % (95% CI)	Thresholds
NIHSS + glucose	46.3 (35.8–56.8)	90.3 (85.5–95.1)	NIHSS ≥ 13; Glucose < 132.5, panel positive when > = 2criteria are met Youden; 0.37	43.5 (32.9–54)	90.6 (85.7–95.4)	NIHSS ≥ 13; Glucose < 132.5 panel positive when ≥2 criteria are met Youden: 0.34
NIHSS + mean blood pressure	47.6 (37.1–58.1)	91.2 (86.4–96.0)	NIHSS ≥ 11, Mean Blood Pressure < 107.833, panel positive when > = 2 criteria are met. Youden: 0.39	32.9 (24.3–42.8)	91.1 (86.3–94.3)	NIHSS ≥ 18; Mean blood pressure >87 panel positive when ≥2 criteria are met. Youden: 0.24
Cincinnati + glucose	25.0 (14.5–35.5)	91.0 (86.2–95.8)	Cincinnati > 2.5, Glucose < 112.5, panel positive when > = 2 criteria are met. Youden: 0.16	23.8 (13.3–34.3)	90.6 (85.8–95.4)	Cincinnati > 2.5, glucose < 112.5, panel positive when > = 2 criteria are met. Youden: 0.14
Cincinnati + mean blood pressure	39.4 (28.9–49.9)	90.5 (85.7–95.3)	Cincinnati > 2.5, mean blood pressure < 107.167, panel positive when > = 2 criteria are met. Youden: 0.30	24.1 (13.6–34.6)	90.2 (85.7–95.3)	Cincinnati > 2.5, mean blood pressure < 103.833, panel positive when > = 2 criteria are met. Youden: 0.14

While these models maintained acceptable specificities, the sensitivity remained substantially lower than that of the biomarker–clinical panel, supporting the incremental contribution of circulating biomarkers. Cross-validated performance metrics for all evaluated panels are provided in Supplementary Table S1.

#### 3.4.3 Panel performance within 6-h from symptom onset

To assess temporal robust ness, the same variable combination was evaluated in patients presenting within 6 h of symptom onset, a clinically relevant therapeutic window. Demographic characteristics of this subgroup are shown in Supplementary Table S2.

After re-estimating the thresholds within this subgroup, the resulting decision rule applied the following cut-offs: NT-proBNP > 564.1 pg./mL, D-dimer > 399.4 ng/mL, mean blood pressure > 79.5 mmHg, and NIHSS ≥ 8. A case was classified as LVO when all four criteria were met. In this 6-h cohort, the panel achieved a sensitivity of 65.7% at a specificity of 90.3% (Table 4). The cross-validated ROC AUC for the model was 0.78, with a normalized cross-validated partial ROC-AUC for the region with specificities above 80% of 0.73.

Across both time windows, the combination of NT-proBNP, D-dimer, mean blood pressure, and NIHSS demonstrated consistent cross-validation performance, with slightly improved sensitivity in the 6-h subgroup while maintaining high specificity in both analyses. These findings suggest only modest optimism bias, as expected in the exploratory setting of this single-cohort study. However, because the 6-h subgroup was nested within the 24-h cohort, these results should be interpreted as analyses of temporal consistency rather than as an independent validation.

#### 3.4.4 Substitution of NIHSS by the Cincinnati prehospital stroke scale

To explore pre-hospital applicability, we evaluated models using the Cincinnati Prehospital Stroke Scale instead of NIHSS. Under the same high-specificity constraint, the model with the optimal biomarker clinical configuration (Cincinnati, mean blood pressure, NT-proBNP and D-dimer) achieved a sensitivity of 56.1% at a specificity of 90.4% in the 6-h cohort (Table 4). In such case the clinical-only model combining Cincinnati Prehospital Stroke Scale and mean blood pressure, but excluding the circulating biomarkers, in the 6-h cohort achieved a sensitivity of 39.4% for a specificity of 90.5%. For the case of a clinical-only model combining Cincinnati Prehospital Stroke Scale and glucose, but excluding circulating biomarkers, in the 6-h cohort achieved a sensitivity of 25.0% for a specificity of 91.0%. Detailed performance metrics for the configuration with Cincinnati Prehospital Stroke Scale are reported in Table 5.

## Discussion

In this study, we identified and internally validated a multivariable panel combining NT-proBNP, D-dimer, mean blood pressure, and NIHSS for the identification of LVO in acute ischemic stroke. Under predefined high-specificity constraints (≥90%), the multimodal configuration outperformed individual biomarkers and clinical variables. When evaluated individually, neither circulating biomarkers nor clinical variables achieved adequate sensitivity at high specificity. Although NT-proBNP and D-dimer levels were significantly elevated in patients with LVO, their discriminative performance remained limited when used in isolation. These findings are consistent with the multifactorial pathophysiology of LVO, which involves heterogeneous mechanisms including cardioembolism and thrombus formation (22). The biological and clinical heterogeneity of LVO likely limits the utility of single-marker strategies and supports the development of multimodal approaches capturing complementary pathophysiological dimensions.

Prehospital clinical scales remain central to stroke triage; however, systematic reviews and meta-analyses have demonstrated variable diagnostic accuracy, particularly when high specificity is required to minimize inappropriate bypass of primary stroke centers. (6, 23). In our cohort, panels restricted to clinical variables alone showed inferior sensitivity under identical high-specificity constraints compared with the biomarker–clinical configuration, reinforcing the limitations of relying solely on neurological examination for LVO detection.

Recent multimodal strategies combining D-dimer and GFAP with prehospital scales such as FAST-ED have reported encouraging diagnostic performance for LVO identification (20, 24–26). However, differences in clinical scale design, cohort characteristics, and optimization strategies limit direct comparisons across studies. In our cohort, comparable configurations incorporating D-dimer and GFAP under identical high-specificity constraints showed lower performance than the NT-proBNP and D-dimer–based panel identified in the present study. Cross-validated performance metrics for all evaluated panels are provided in Supplementary Table S1. This observation suggests that inclusion of markers reflecting cardioembolic mechanisms may provide incremental discriminatory value in LVO detection beyond thrombotic and structural injury markers alone.

Using a data-driven approach with PanelomiX, we derived a biologically coherent panel integrating cardiac stress (NT-proBNP), coagulation activation (D-dimer), hemodynamic status (mean blood pressure), and neurological severity (NIHSS). Together, these parameters capture complementary dimensions of LVO pathophysiology and likely explain the improved classification performance observed. Notably, although age and sex differed significantly between groups, they were not retained in the final model, suggesting limited incremental predictive value once pathophysiological markers were incorporated.

The panel also showed consistent performance within the 6-h therapeutic window, which is particularly relevant for mechanical thrombectomy decision-making. In this subgroup, the same panel components were retained and achieved slightly higher sensitivity while maintaining high specificity. Threshold values were largely stable across onset time windows, with only a small shift in NT-proBNP while the remaining thresholds remained unchanged, supporting the stability of the identified configuration. However, because the 6-h subgroup was nested within the 24-h cohort rather than representing an independent population, these findings should be interpreted as analyses of temporal consistency rather than as an independent external validation.

To explore real-world applicability, we replaced NIHSS with the Cincinnati Prehospital Stroke Scale within the same biomarker–clinical framework. While NIHSS is a comprehensive and validated neurological scale, its use in prehospital EMS settings may be limited by time constraints and the need for trained personnel. Although sensitivity was decreased, specificity remained preserved. Given the simplicity and widespread implementation of Cincinnati in prehospital settings, these findings suggest that circulating biomarkers may compensate for the reduced granularity of simplified neurological scales and facilitate pragmatic integration into emergency workflows.

Rather than replacing imaging-based diagnosis, this biomarker–clinical panel should be viewed as a decision-support tool for triage within established hub-and-spoke stroke networks. The panel was intentionally optimized for high specificity and is therefore best interpreted as a rule-in enrichment strategy, identifying a subgroup of patients with a high probability of LVO who may benefit from direct routing to thrombectomy-capable centers. It was not designed as a rule-out strategy for excluding LVO in patients classified as low risk. This distinction is clinically important because indiscriminate bypass of primary stroke centers may delay care for non-LVO patients and has not consistently improved outcomes in randomized triage studies such as RACECAT (27). The optimal sensitivity–specificity trade-off is therefore likely to depend on local geography, transfer times, thrombolysis access, thrombectomy availability, and stroke-network organization.

Several limitations warrant consideration. First, this panel was derived within a single prospective cohort and requires external validation in independent populations. We fully acknowledge that the present results should be considered exploratory and hypothesis-generating until externally validated. Although cross-validation was applied for panel ranking and robustness assessment, prospective validation remains essential before clinical implementation. Comparing apparent performance estimations with cross-validated metrics (Supplementary Table S1) signaled modest optimism bias as expected with the exploratory design of this single-cohort study. Nevertheless, optimized cut-off values may vary across populations and healthcare systems, and larger multicenter studies will be necessary to confirm the robustness and generalizability of these findings.

Second, biomarker measurements were obtained under controlled laboratory conditions, and performance in real-world prehospital environments remains to be established. Future studies should therefore evaluate this strategy using dedicated point-of-care (POC) devices integrated into operational EMS workflows. Portable POC devices for D-dimer and NT-proBNP are already commercially available, supporting the feasibility of biomarker-based prehospital triage. The panel identified here is currently being implemented within a dedicated POC platform for prospective evaluation in EMS settings, including assessment of analytical reliability, turnaround time, and operational integration.

Third, missing data were handled by design using available-case analysis per panel. This approach was chosen *a priori* over complete-case analysis, which would have excluded 30% of the cohort, fundamentally altering cohort composition and introducing selection bias unrelated to the analytical question. To assess robustness, a sensitivity analysis was conducted applying stricter exclusion criteria for all variables except GFAP, given its disproportionate missingness (15.9%), yielding a cohort of 257 patients where non-GFAP panels experienced an exclusion rate comparable to that of GFAP-containing panels in the primary analysis. This comparison confirmed identical panel composition for the top ranked panels, and consistent cross-validated performances within current uncertainties, with minor differences attributable to stochastic variations across cross-validation.

Nevertheless, although the available-case approach preserved sample size and avoided excluding patients with missing values in variables not included in a given panel, residual bias cannot be excluded if missingness was not completely random. Accordingly, the impact of missing biomarker data should be reassessed in larger prospective cohorts with predefined sampling procedures and external validation.

The impact of missing biomarker data on panel performance, as well as the contribution of categorical clinical history variables, larger panel configurations, and alternative multivariate approaches all warrant further investigation in larger prospective cohorts.

In conclusion, while individual circulating biomarkers demonstrate limited standalone diagnostic utility, their integration with simple clinical parameters yields a biologically coherent and cross-validated multivariable panel for LVO identification under high-specificity constraints. Multimodal strategies that reflect the complex pathophysiology of LVO may represent a pragmatic path toward safer and more effective early stroke triage.

## Data Availability

The datasets presented in this study can be found in online repositories. The names of the repository/repositories and accession number(s) can be found in the article/Supplementary material.
