# Supplementary material for: High-specificity identification of large vessel occlusion stroke using D-dimer and NT-proBNP combined with clinical variables
Source: Front Neurol. 2026 Jun 24;17:1830051. doi: 10.3389/fneur.2026.1830051 (PMC13341300; doi:10.3389/fneur.2026.1830051)
Supplement: Supplementary file 1 [file Data_Sheet_1.docx]

Supplementary Material

# Supplementary Table 1

Supplementary Table 1: Cross-validated diagnostic performance of all evaluated multivariable panels for large vessel occlusion identification in the 24h population.

| Panel | CV-spec | CV-sens | CV-Youden | CV-pAUC (>0.8) | CV-pAUC(>0.9) | CV-AUC |
| --- | --- | --- | --- | --- | --- | --- |
| NIHSS+Mean Blood Pressure+D-Dimer+NT-proBNP | 0.888 | 0.548 | 0.436 | 0.678 | 0.629 | 0.764 |
| NIHSS+Mean Blood Pressure+NT-proBNP+H-FABP | 0.884 | 0.517 | 0.401 | 0.661 | 0.618 | 0.742 |
| NIHSS+Mean Blood Pressure+NT-proBNP | 0.891 | 0.489 | 0.379 | 0.658 | 0.619 | 0.738 |
| NIHSS+Sex+Mean Blood Pressure+NT-proBNP | 0.860 | 0.499 | 0.359 | 0.641 | 0.602 | 0.729 |
| NIHSS+Age+Glucosa+D-Dimer | 0.858 | 0.498 | 0.356 | 0.639 | 0.599 | 0.716 |
| NIHSS+Mean Blood Pressure+Age+NT-proBNP | 0.871 | 0.476 | 0.346 | 0.646 | 0.609 | 0.731 |
| NIHSS+Glucosa | 0.895 | 0.436 | 0.331 | 0.642 | 0.616 | 0.683 |
| NIHSS+Age+Glucosa | 0.885 | 0.445 | 0.330 | 0.632 | 0.603 | 0.687 |
| NIHSS+Age+Glucosa+H-FABP | 0.871 | 0.459 | 0.330 | 0.631 | 0.601 | 0.688 |
| NIHSS+NT-proBNP+H-FABP | 0.885 | 0.434 | 0.319 | 0.633 | 0.596 | 0.719 |
| NIHSS+Mean Blood Pressure+Age+D-Dimer | 0.860 | 0.456 | 0.316 | 0.623 | 0.588 | 0.713 |
| NIHSS+Mean Blood Pressure+NT-proBNP+GFAP | 0.882 | 0.431 | 0.313 | 0.634 | 0.597 | 0.723 |
| NIHSS+NT-proBNP | 0.892 | 0.417 | 0.309 | 0.633 | 0.592 | 0.730 |
| NIHSS+Age+D-Dimer | 0.885 | 0.423 | 0.308 | 0.626 | 0.592 | 0.701 |
| NIHSS+Glucosa+H-FABP | 0.880 | 0.425 | 0.305 | 0.629 | 0.605 | 0.672 |
| NIHSS+Sex+Age+D-Dimer | 0.866 | 0.436 | 0.302 | 0.618 | 0.584 | 0.684 |
| NIHSS+Mean Blood Pressure+D-Dimer+H-FABP | 0.871 | 0.431 | 0.301 | 0.621 | 0.580 | 0.720 |
| NIHSS+Mean Blood Pressure+Glucosa+NT-proBNP | 0.856 | 0.442 | 0.299 | 0.618 | 0.585 | 0.706 |
| NIHSS+Age+D-Dimer+H-FABP | 0.869 | 0.427 | 0.297 | 0.621 | 0.583 | 0.699 |
| NIHSS+Age+Glucosa+NT-proBNP | 0.846 | 0.450 | 0.296 | 0.609 | 0.575 | 0.682 |
| NIHSS+Mean Blood Pressure+Age+Glucosa | 0.838 | 0.456 | 0.294 | 0.615 | 0.584 | 0.687 |
| NIHSS+Sex+NT-proBNP | 0.875 | 0.416 | 0.291 | 0.618 | 0.581 | 0.717 |
| NIHSS+Age+Glucosa+GFAP | 0.859 | 0.430 | 0.289 | 0.609 | 0.577 | 0.676 |
| NIHSS+Mean Blood Pressure+Glucosa | 0.873 | 0.411 | 0.284 | 0.619 | 0.595 | 0.673 |
| NIHSS+Sex+D-Dimer+NT-proBNP | 0.856 | 0.427 | 0.284 | 0.607 | 0.563 | 0.71 |
| NIHSS+Mean Blood Pressure+D-Dimer | 0.865 | 0.418 | 0.282 | 0.610 | 0.575 | 0.703 |
| NIHSS+NT-proBNP+GFAP | 0.873 | 0.409 | 0.281 | 0.617 | 0.579 | 0.719 |
| NIHSS+D-Dimer+GFAP | 0.881 | 0.395 | 0.276 | 0.615 | 0.587 | 0.690 |
| NIHSS+D-Dimer | 0.886 | 0.388 | 0.274 | 0.614 | 0.585 | 0.693 |
| NIHSS+Age+NT-proBNP | 0.873 | 0.400 | 0.273 | 0.612 | 0.572 | 0.720 |
| NIHSS+Age | 0.887 | 0.378 | 0.265 | 0.618 | 0.586 | 0.699 |
| NIHSS+Sex+D-Dimer | 0.875 | 0.39 | 0.265 | 0.605 | 0.572 | 0.683 |
| NIHSS+D-Dimer+NT-proBNP+H-FABP | 0.844 | 0.420 | 0.264 | 0.604 | 0.570 | 0.699 |
| NIHSS+Mean Blood Pressure+Glucosa+D-Dimer | 0.847 | 0.416 | 0.263 | 0.604 | 0.569 | 0.708 |
| NIHSS+Mean Blood Pressure+Age | 0.868 | 0.394 | 0.262 | 0.604 | 0.573 | 0.695 |
| NIHSS+Mean Blood Pressure+D-Dimer+GFAP | 0.859 | 0.398 | 0.257 | 0.606 | 0.573 | 0.707 |
| NIHSS+Sex+Mean Blood Pressure+Glucosa | 0.833 | 0.423 | 0.256 | 0.607 | 0.575 | 0.703 |
| NIHSS+Age+GFAP | 0.861 | 0.394 | 0.255 | 0.613 | 0.584 | 0.709 |
| NIHSS+Glucosa+GFAP | 0.863 | 0.390 | 0.253 | 0.603 | 0.575 | 0.664 |
| NIHSS+Glucosa+H-FABP+GFAP | 0.853 | 0.400 | 0.253 | 0.604 | 0.578 | 0.654 |
| NIHSS+Sex+Glucosa+NT-proBNP | 0.847 | 0.406 | 0.253 | 0.609 | 0.574 | 0.715 |
| NIHSS+Mean Blood Pressure+Age+GFAP | 0.862 | 0.387 | 0.249 | 0.601 | 0.564 | 0.697 |
| NIHSS+Mean Blood Pressure+H-FABP | 0.878 | 0.371 | 0.248 | 0.599 | 0.570 | 0.654 |
| NIHSS+Mean Blood Pressure+Glucosa+GFAP | 0.847 | 0.401 | 0.248 | 0.601 | 0.574 | 0.669 |
| NIHSS+Mean Blood Pressure+Glucosa+H-FABP | 0.844 | 0.402 | 0.245 | 0.592 | 0.565 | 0.654 |
| NIHSS+Glucosa+NT-proBNP | 0.855 | 0.387 | 0.242 | 0.593 | 0.562 | 0.672 |
| NIHSS+Glucosa+D-Dimer | 0.849 | 0.392 | 0.241 | 0.597 | 0.569 | 0.696 |
| NIHSS+D-Dimer+H-FABP | 0.862 | 0.379 | 0.241 | 0.597 | 0.563 | 0.679 |
| NIHSS+Sex+Mean Blood Pressure+Age | 0.859 | 0.382 | 0.241 | 0.601 | 0.568 | 0.71 |
| NIHSS+Age+D-Dimer+GFAP | 0.855 | 0.386 | 0.241 | 0.601 | 0.571 | 0.678 |
| NIHSS+Age+NT-proBNP+H-FABP | 0.853 | 0.388 | 0.240 | 0.603 | 0.573 | 0.695 |
| NIHSS+Sex+Age+NT-proBNP | 0.854 | 0.384 | 0.239 | 0.593 | 0.559 | 0.695 |
| NIHSS+Age+H-FABP+GFAP | 0.865 | 0.373 | 0.238 | 0.602 | 0.574 | 0.697 |
| NIHSS+Sex+Glucosa+D-Dimer | 0.849 | 0.389 | 0.238 | 0.599 | 0.566 | 0.691 |
| NIHSS+Sex+Mean Blood Pressure | 0.854 | 0.381 | 0.235 | 0.591 | 0.561 | 0.664 |
| NIHSS+Mean Blood Pressure+Age+H-FABP | 0.848 | 0.387 | 0.235 | 0.596 | 0.556 | 0.711 |
| NIHSS+Mean Blood Pressure | 0.885 | 0.348 | 0.232 | 0.592 | 0.565 | 0.620 |
| NIHSS+Glucosa+D-Dimer+H-FABP | 0.816 | 0.413 | 0.230 | 0.593 | 0.562 | 0.687 |
| NIHSS+Age+D-Dimer+NT-proBNP | 0.854 | 0.374 | 0.228 | 0.592 | 0.560 | 0.678 |
| NIHSS+Sex+NT-proBNP+GFAP | 0.840 | 0.388 | 0.228 | 0.601 | 0.574 | 0.69 |
| NIHSS+Glucosa+NT-proBNP+H-FABP | 0.825 | 0.398 | 0.223 | 0.583 | 0.554 | 0.675 |
| NIHSS+D-Dimer+H-FABP+GFAP | 0.864 | 0.359 | 0.223 | 0.593 | 0.568 | 0.677 |
| NIHSS+Age+H-FABP | 0.855 | 0.366 | 0.221 | 0.589 | 0.562 | 0.663 |
| NIHSS+Sex+NT-proBNP+H-FABP | 0.818 | 0.403 | 0.22 | 0.591 | 0.559 | 0.688 |
| NIHSS+Sex+Mean Blood Pressure+D-Dimer | 0.827 | 0.392 | 0.219 | 0.583 | 0.554 | 0.69 |
| NIHSS+Sex+Age+GFAP | 0.815 | 0.399 | 0.214 | 0.595 | 0.565 | 0.69 |
| NIHSS+Sex | 0.881 | 0.332 | 0.213 | 0.598 | 0.567 | 0.708 |
| NIHSS+Glucosa+D-Dimer+NT-proBNP | 0.822 | 0.391 | 0.213 | 0.580 | 0.552 | 0.677 |
| NIHSS+Glucosa+NT-proBNP+GFAP | 0.822 | 0.391 | 0.213 | 0.575 | 0.550 | 0.658 |
| NIHSS+Sex+Age+Glucosa | 0.84 | 0.369 | 0.209 | 0.592 | 0.57 | 0.684 |
| NIHSS+NT-proBNP+H-FABP+GFAP | 0.842 | 0.366 | 0.208 | 0.589 | 0.562 | 0.692 |
| NIHSS+Mean Blood Pressure+GFAP | 0.867 | 0.333 | 0.199 | 0.574 | 0.550 | 0.622 |
| NIHSS+D-Dimer+NT-proBNP | 0.852 | 0.341 | 0.193 | 0.585 | 0.557 | 0.675 |
| NIHSS+Sex+Age | 0.86 | 0.331 | 0.191 | 0.572 | 0.544 | 0.675 |
| NIHSS+Sex+D-Dimer+GFAP | 0.813 | 0.377 | 0.19 | 0.577 | 0.55 | 0.672 |
| NIHSS+Sex+Glucosa | 0.846 | 0.339 | 0.184 | 0.578 | 0.558 | 0.652 |
| NIHSS+Glucosa+D-Dimer+GFAP | 0.802 | 0.382 | 0.183 | 0.561 | 0.534 | 0.653 |
| NIHSS+Sex+D-Dimer+H-FABP | 0.844 | 0.339 | 0.182 | 0.567 | 0.541 | 0.65 |
| NIHSS+Sex+Age+H-FABP | 0.838 | 0.339 | 0.177 | 0.573 | 0.544 | 0.657 |
| NIHSS+Age+NT-proBNP+GFAP | 0.816 | 0.359 | 0.175 | 0.575 | 0.545 | 0.686 |
| NIHSS+Sex+Mean Blood Pressure+H-FABP | 0.812 | 0.36 | 0.172 | 0.57 | 0.545 | 0.663 |
| NIHSS+Sex+Glucosa+GFAP | 0.813 | 0.358 | 0.171 | 0.568 | 0.55 | 0.653 |
| NIHSS+Sex+Mean Blood Pressure+GFAP | 0.816 | 0.353 | 0.169 | 0.56 | 0.539 | 0.65 |
| NIHSS+D-Dimer+NT-proBNP+GFAP | 0.802 | 0.358 | 0.161 | 0.571 | 0.545 | 0.673 |
| NIHSS+H-FABP | 0.836 | 0.321 | 0.158 | 0.569 | 0.546 | 0.640 |
| NIHSS+Mean Blood Pressure+H-FABP+GFAP | 0.852 | 0.306 | 0.158 | 0.560 | 0.540 | 0.615 |
| NIHSS+Sex+Glucosa+H-FABP | 0.834 | 0.319 | 0.153 | 0.568 | 0.551 | 0.652 |
| NIHSS+Sex+GFAP | 0.83 | 0.32 | 0.15 | 0.578 | 0.551 | 0.681 |
| NIHSS+Sex+H-FABP | 0.824 | 0.324 | 0.147 | 0.57 | 0.545 | 0.678 |
| NIHSS+GFAP | 0.830 | 0.314 | 0.144 | 0.565 | 0.553 | 0.611 |
| NIHSS+H-FABP+GFAP | 0.825 | 0.311 | 0.136 | 0.565 | 0.548 | 0.629 |
| Age | 0.905 | 0.224 | 0.129 | 0.556 | 0.546 | 0.565 |
| NIHSS+Sex+H-FABP+GFAP | 0.786 | 0.306 | 0.092 | 0.547 | 0.53 | 0.634 |
| NIHSS | 0.899 | 0.185 | 0.084 | 0.537 | 0.532 | 0.542 |
| D-dimer | 0.941 | 0.092 | 0.033 | 0.516 | 0.515 | 0.517 |
| NT-proBNP | 0.900 | 0.128 | 0.027 | 0.514 | 0.513 | 0.514 |
| H-FABP | 0.908 | 0.117 | 0.025 | 0.517 | 0.518 | 0.512 |
| Glucosa | 0.902 | 0.070 | -0,028 | 0.495 | 0.502 | 0.486 |
| GFAP | 0.940 | 0.000 | -0,06 | 0.485 | 0.493 | 0.470 |
| Mean Blood Pressure | 0.901 | 0.011 | -0,088 | 0.474 | 0.486 | 0.456 |

Supplementary table 2: Demographic and clinical characteristics of the 6-hour population according to LVO status

|  | **No LVO** | **LVO** | **Overall** | **P-value** |
| --- | --- | --- | --- | --- |
|  | **(N=152)** | **(N=71)** | **(N=223)** |  |
| **Age (years)** |  |  |  | <0.001 |
| Mean (SD) | 69.3 (12.0) | 75.9 (10.1) | 71.4 (11.8) |  |
| Median [Min, Max] | 70.0 [34.0, 93.0] | 76.0 [52.0, 95.0] | 73.0 [34.0, 95.0] |  |
| **Sex** |  |  |  |  |
| Male | 1.36 (0.480) | 1.52 (0.503) | 1.41 (0.493) | 0.064 |
| Female | 1.00 [1.00, 2.00] | 2.00 [1.00, 2.00] | 1.00 [1.00, 2.00] |  |
| **NIHSS** |  |  |  |  |
| Mean (SD) | 8.73 (9.53) | 15.4 (7.24) | 10.9 (9.37) | <0.001 |
| Median [Min, Max] | 5.00 [0, 35.0] | 16.0 [1.00, 35.0] | 8.00 [0, 35.0] |  |
| **Systolic Blood Pressure (mmHg)** | |  |  |  |
| Mean (SD) | 154 (33.4) | 152 (23.4) | 153 (30.5) | 0.996 |
| Median [Min, Max] | 150 [80.0, 260] | 150 [96.0, 220] | 150 [80.0, 260] |  |
| **Diastolic Blood Pressure (mmHg)** | |  |  |  |
| Mean (SD) | 86.3 (20.0) | 84.5 (12.0) | 85.7 (17.8) | 0.841 |
| Median [Min, Max] | 87.0 [34.0, 171] | 84.0 [55.0, 111] | 86.0 [34.0, 171] |  |
| **Mean Blood Pressure (mmHg)** |  |  |  |  |
| Mean (SD) | 101 (35.6) | 101 (27.8) | 101 (33.2) | 0.987 |
| Median [Min, Max] | 107 [0, 182] | 107 [0, 133] | 107 [0, 182] |  |
| **Diabetes Mellitus** |  |  |  |  |
| No | 1.32 (0.469) | 1.28 (0.453) | 1.31 (0.463) | 0.829 |
| Yes | 1.00 [1.00, 2.00] | 1.00 [1.00, 2.00] | 1.00 [1.00, 2.00] |  |
| **Atrial Fibrillation** |  |  |  |  |
| No | 124 (81.6%) | 49 (69.0%) | 173 (77.6%) | 0.111 |
| Yes | 28 (18.4%) | 22 (31.0%) | 50 (22.4%) |  |
| **Heart rate (beats/min)** |  |  |  |  |
| Mean (SD) | 80.7 (18.5) | 77.9 (16.9) | 79.8 (18.0) | 0.661 |
| Median [Min, Max] | 78.0 [35.0, 140] | 75.0 [44.0, 140] | 76.0 [35.0, 140] |  |
| **Glucose (mg/dL)** |  |  |  |  |
| Mean (SD) | 139 (72.3) | 133 (55.5) | 137 (67.3) | 0.905 |
| Median [Min, Max] | 121 [66.0, 750] | 116 [82.0, 382] | 119 [66.0, 750] |  |
| **Smoking** |  |  |  |  |
| Former smoker | 39 (25.7%) | 16 (22.5%) | 55 (24.7%) | 0.968 |
| Never smoker | 84 (55.3%) | 43 (60.6%) | 127 (57.0%) |  |
| Current smoker | 29 (19.1%) | 12 (16.9%) | 41 (18.4%) |  |

Data are presented as mean (SD), median [min–max], or number (%), as appropriate. P-values were calculated using Student’s t-test, Mann–Whitney U test, or χ² test, as appropriate.

Supplementary figure 1: Study workflow and diagnostic composition of the study cohorts.
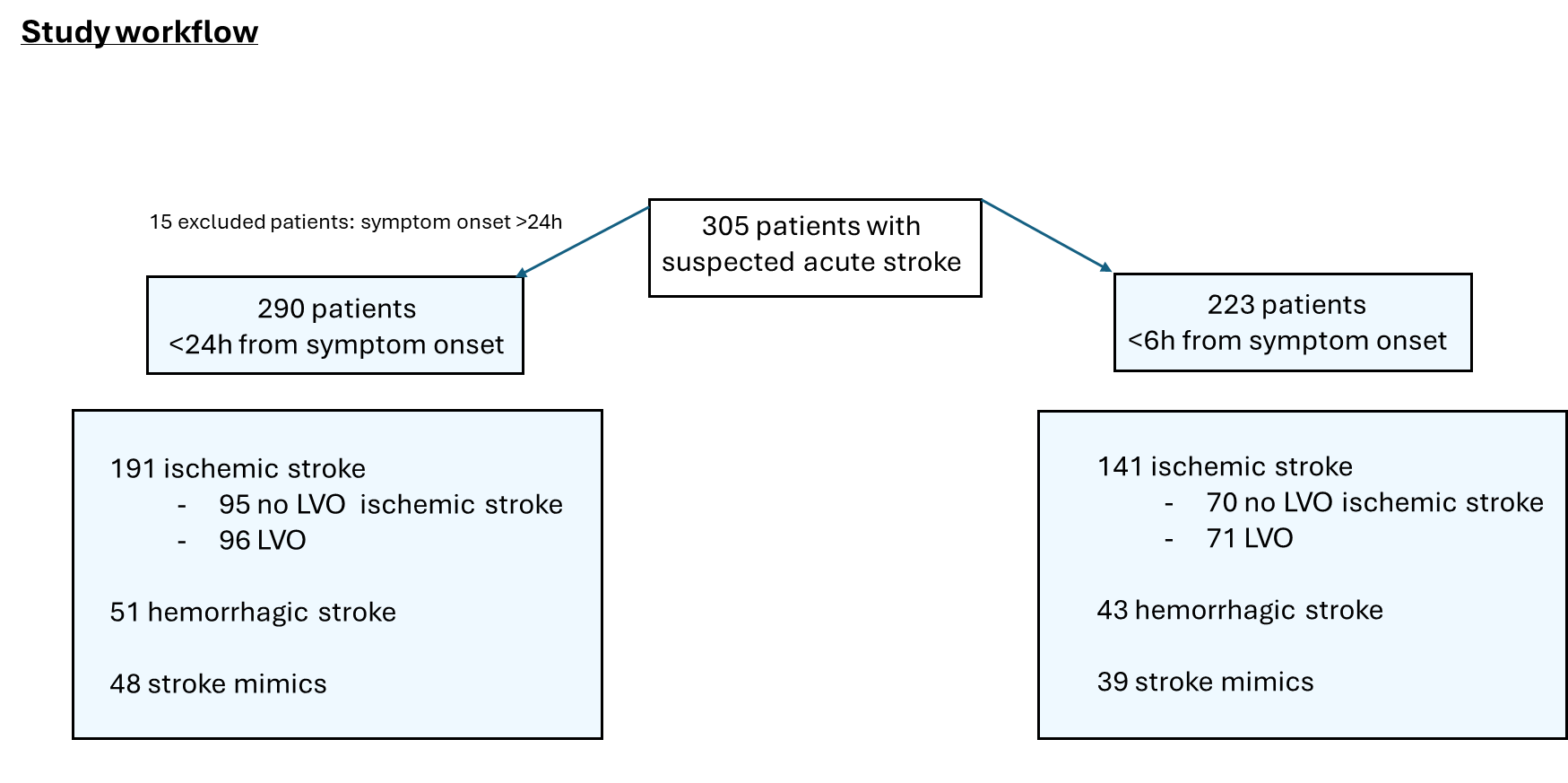


Flowchart summarizing patient selection and diagnostic classification in the ≤24 h and ≤6 h cohorts. The non-LVO group included non-LVO ischemic stroke, intracerebral hemorrhage, and stroke mimics. LVO: large vessel occlusion.

Supplementary figure 2: Diagnostic performance of individual predictors and the final multimodal panel for large vessel occlusion detection at fixed high specificity


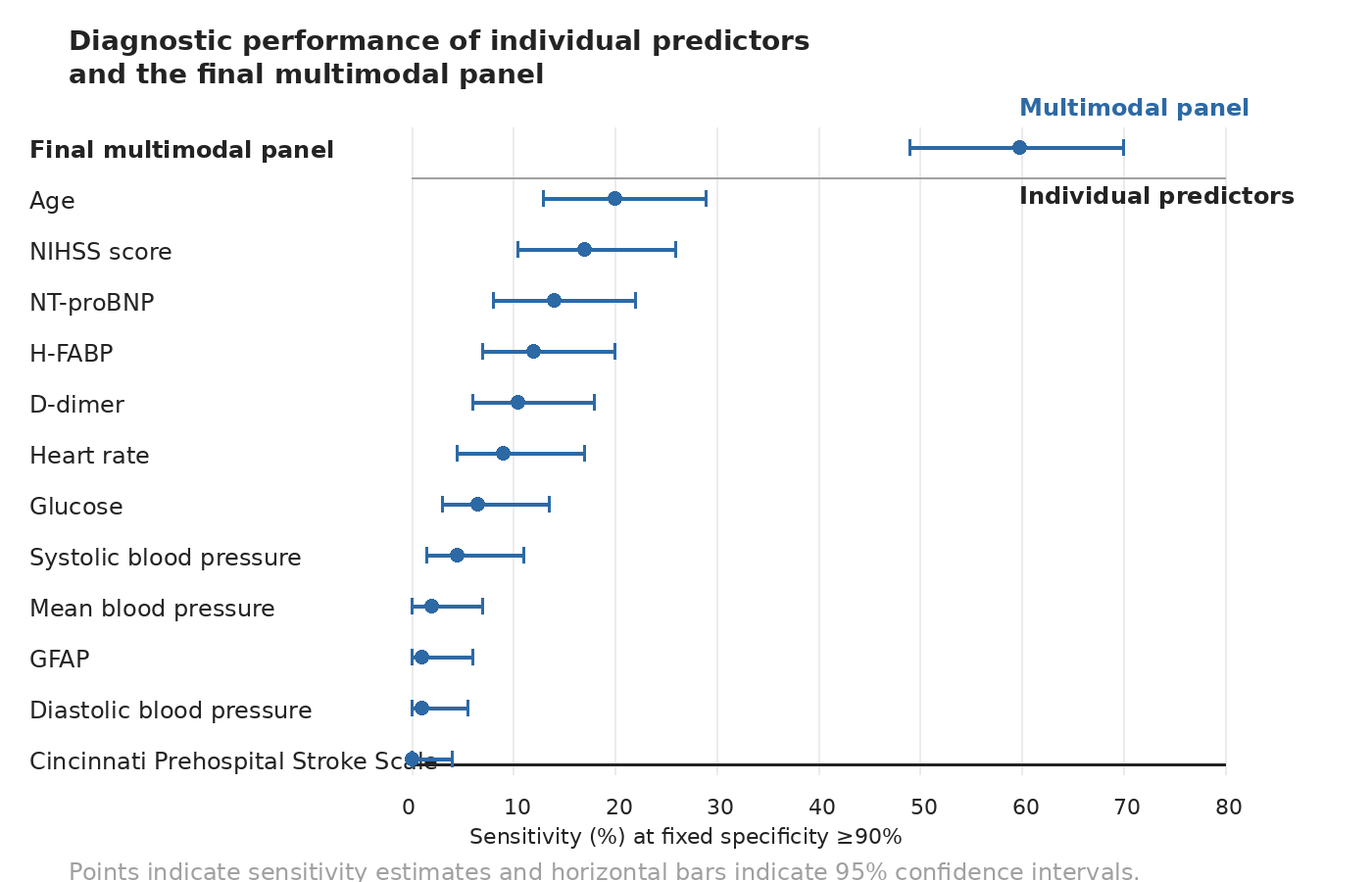


Sensitivity estimates of individual clinical and biomarker predictors, as well as the final multimodal panel, evaluated at a fixed specificity threshold ≥90% in the ≤24 h cohort. Points represent sensitivity estimates and horizontal bars indicate 95% confidence intervals. The final multimodal panel combined NIHSS score, mean blood pressure, D-dimer, and NT-proBNP. LVO: large vessel occlusion; NIHSS: National Institutes of Health Stroke Scale; NT-proBNP: N-terminal pro–B-type natriuretic peptide; H-FABP: heart-type fatty acid-binding protein; GFAP: glial fibrillary acidic protein.

**
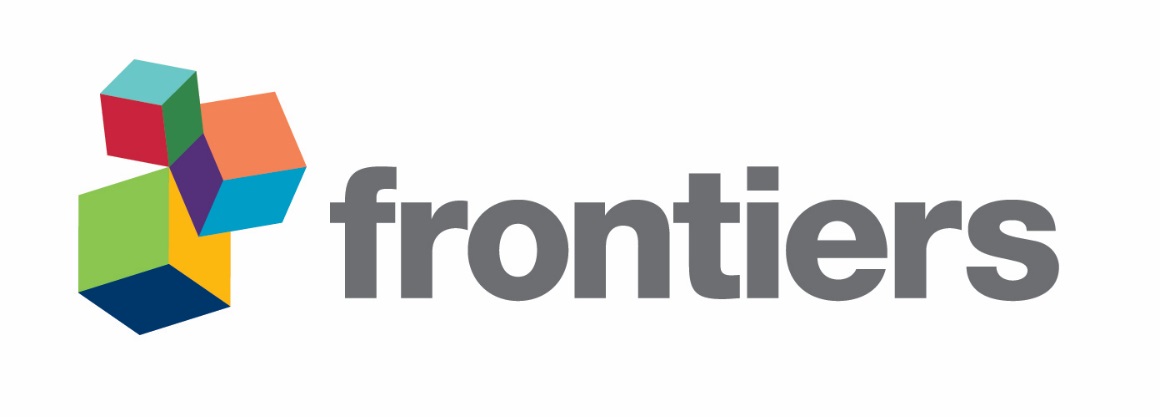
**
